# The rs140668532 SNP in GSK-3β gene as a potential biomarker for Alzheimer’s disease: Insights from computational modeling

**DOI:** 10.1016/j.toxrep.2025.102060

**Published:** 2025-05-28

**Authors:** İrem Gülfem Albayrak, Belkıs Atasever Arslan

**Affiliations:** Department of Molecular Biology and Genetics, Faculty of Engineering and Natural Sciences, Üsküdar University, Istanbul, Turkey

**Keywords:** GSK-3β, SNPs, Alzheimer's disease, Tau, Molecular docking

## Abstract

Glycogen synthase kinase-3 beta (GSK-3β) is well recognized for its role in diverse physiological processes, including apoptosis, mitochondrial function, and gene transcription regulation. The precise regulation of GSK-3β activity is critical for maintaining neuronal health, and dysregulation may result in disturbances in neurological functions. Polymorphisms in the GSK-3β gene may increase susceptibility to neurodegenerative disorders. To assess the structural and functional consequences of deleterious SNPs in GSK-3β, various in silico approaches was utilized. Analysis identified 27 deleterious SNPs in the GSK-3β gene, among which 10 were classified as damaging by SIFT, PolyPhen-2, and MutPred2. The Project Hope software simulated ten harmful mutations in the GSK-3β gene. The pathways associated with neurodegeneration involving the GSK-3β gene and its interacting genes were identified through the KEGG and GeneMANIA databases, respectively. The V317F mutation was shown to reduce GSK-3β inhibition by highly selective inhibitory ligand PF04802367 (PF-367) and impair the GSK-3β–Tau interaction. The influence of GSK3β on Aβ formation suggests that the V317F mutation has a tau-independent neurodegenerative impact. The experimental investigation of the V317F mutant GSK-3β's effect on neurodegeneration may enhance the understanding of the biomarker potential of rs140668532 in Alzheimer's disease.

## Highlights

• Twenty-seven deleterious SNPs have been identified in the GSK-3β gene.
• Numerous in silico approaches have been applied to analyze the effects of these SNPs.
• rs140668532 reduced GSK-3β inhibition and weakened its interaction with Tau protein.
• The rs140668532 SNP may be a potential biomarker in Alzheimer's disease.

## Graphical Abstract

**Figure:**
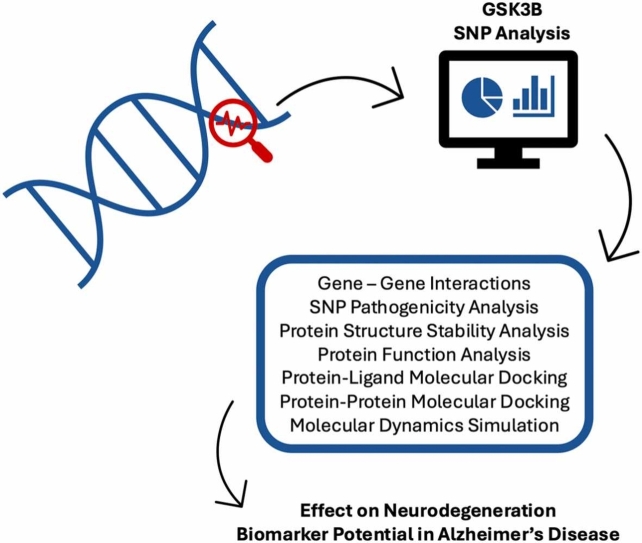

## 1 Introduction

Glycogen synthase kinase-3 (GSK-3) is a kinase that has been well conserved throughout evolution [1], [2], [3]. Extensive studies have linked GSK-3 to the regulation of numerous essential physiological processes, identifying more than 40 distinct proteins as its substrates [3], [4]. Two highly homologous forms of GSK-3, GSK-3α and GSK-3β, have distinct N-terminal regions but are 98 % similar in the internal kinase domain and have strong expression in the brain [5], [6], [7], [8]. GSK-3β regulates glycogen production to control glycogen consumption, modulates mitochondrial permeability, and facilitates cytochrome C release to control apoptosis [9], [10]. Highly expressed in neural tissues, GSK-3β has been implicated in several neurological disorders, including Alzheimer’s disease (AD), Parkinson’s disease (PD), and schizophrenia [11], [12], [13]. Notably, inhibition of GSK3β in neuronal cells has been displayed as protecting neuroprogenitor cells against genotoxicity and other forms of apoptosis triggered by stress [8], [14], [15].

GSK-3 has several regulatory effects on neuronal processes involved in gene transcription, metabolism, apoptosis, and cytoskeletal dynamics. To ensure the precise coordination of these processes, it is essential to meticulously regulate GSK-3 activity through the interplay of phosphorylation, localization, and sequestration by proteins that interact with GSK-3 [3], [4], [16], [17].

Single nucleotide polymorphisms (SNPs) represent the most common type of genetic variation in the human genome. SNPs can increase susceptibility to one or more illnesses by affecting biological pathways. To uncover SNPs that affect disease risk and progression, genotyping is performed in both patient cohorts and control populations, followed by the analysis of significant differences in allele frequencies between the groups. [18]. The identification of SNPs in the GSK-3β gene may have an influence on the molecular pathways associated with neurodegenerative disorders. The present study applied computational techniques, such as SIFT, PolyPhen-2, I-Mutant 2.0, Project HOPE, MutPred2, molecular docking and MD simulation to detect possible functional effects of harmful SNPs in the GSK-3β gene prior to experimental validation.

## 2 Methods

### 2.1 Determining Gene – Gene Interactions and Related Pathways

The GeneMANIA database was utilized to identify gene-gene interactions. Regular updates informed by contemporary research guarantee the accuracy and significance of the data. This study employed an autonomously chosen measuring technique for its analysis. The correlations of pertinent genes with neurodegeneration were assessed utilizing the Genecards and NCBI Gene databases. The KEGG pathway database was applied to ascertain the function of GSK-3β in potential neurodegenerative signaling pathways.

### 2.2 Data Acquisition

SNPs in the GSK3B gene were retrieved from the NCBI dbSNP database in July 2024. Among these SNPs, only missense changes were chosen for further investigation. Missense SNPs were prioritized in this study due to their potential to induce amino acid substitutions, which may directly impact protein structure and function, thereby contributing to disease pathogenesis. The amino acid sequence data associated with this accession number was obtained from the Uniprot database.

### 2.3 Pathogenicity Analysis

The pathogenicity assessment involved the detection of harmful SNPs utilizing the online software tools SIFT (Sorting Intolerant from Tolerant) and PolyPhen2 (Polymorphism Phenotyping V2). The SIFT program integrates sequence homology and the physicochemical characteristics of amino acids to evaluate the effects of amino acid alterations on protein functionality. Amino acid substitutions with values below 0.05 are expected to have adverse consequences or be poorly tolerated by the organism, whereas substitutions with values over 0.05 are regarded as acceptable [19].

PolyPhen-2 is a software tool that forecasts the possible effects of amino acid alterations on the structure and function of human proteins. The PolyPhen score (PSIC score) is computed for genetic variants within each area, as well as the incompatibilities observed among them. A greater disparity in scores across variants indicates that a specific amino acid modification may provide more substantial functional implications [20].

### 2.4 Protein Structure Stability Analysis

The I-Mutant 2.0 tool was used to evaluate the influence of amino acid changes on protein stabilization. A negative free energy ΔΔG (kcal/mol) signifies decreased stability [21].

### 2.5 Protein Function Analysis

Project HOPE is a software program (https://www3.cmbi.umcn.nl/hope/method/). HOPE examines the impact of a specific mutation on protein structure. Modeling enables the assessment of the effects of both wild-type and mutant amino acids on the protein's 3D conformation.

### 2.6 Protein-Ligand Molecular Docking

PF04802367, a selective inhibitor of GSK-3, was used for ligand–protein docking investigations [22]. Its SMILES format was obtained from the PubChem database. The three-dimensional structures of the GSK-3β protein (5K5N) were obtained from the RCSB Protein Data Bank. The BIOVIA Discovery Studio Visualizer 2024 software was employed for molecular docking preparations. The amino acids of 5K5N were initially examined. The modifications included adjustments to CYS317VAL, PRO372LEU, and ASN370PRO, and the pdb file was saved for use as WT. Subsequently, point mutations VAL304PHE, ARG278GLY, ILE182THR, LEU359PHE, PRO357ALA, VAL317PHE, LEU372PHE, and PRO370ALA were introduced into this protein and stored as individual pdb files.

CB-Dock-2 web server was used to determine their docking energy affinities (kcal/mole) [23]. This software facilitates protein-ligand docking by autonomously identifying binding sites (cavities), calculating their centers and dimensions, adjusting the docking box size of the protein drug target based on the query ligands, and subsequently executing molecular docking using AutoDock Vina. The WT GSK3 β–ligand complex exhibiting the lowest binding energy was chosen to analyze alterations in amino acid residues associated with binding, the type of bonds formed, and the bond lengths for each mutation.

### 2.7 Protein-Protein Molecular Docking

Docking investigations of GSK3 β and Tau(267−312) proteins were conducted using the ClusPro2.0 web server [24]. Interactions between wild-type and mutant GSK3 β with Tau(267−312) were shown using the RCSB PDB Mol* 3D Viewer [25].

### 2.8 Molecular Dynamics Simulation

Molecular docking results indicated that the stability evaluation of WT and VAL317PHE mutant GSK3 β receptors for PF04802367 and Tau(267−312) was executed by molecular dynamics simulations utilizing GROMACS with the AMBER99SB-ILDN force field [26], [27]. The study of the MD simulation data involved the computation of many parameters, including the Root Mean Square Deviation (RMSD), Root Mean Square Fluctuation (RMSF), and Radius of Gyration (RoG).

## 3 Results

### 3.1 Gene – Gene Interactions and Pathyway Analysis

**Fig. 1:**
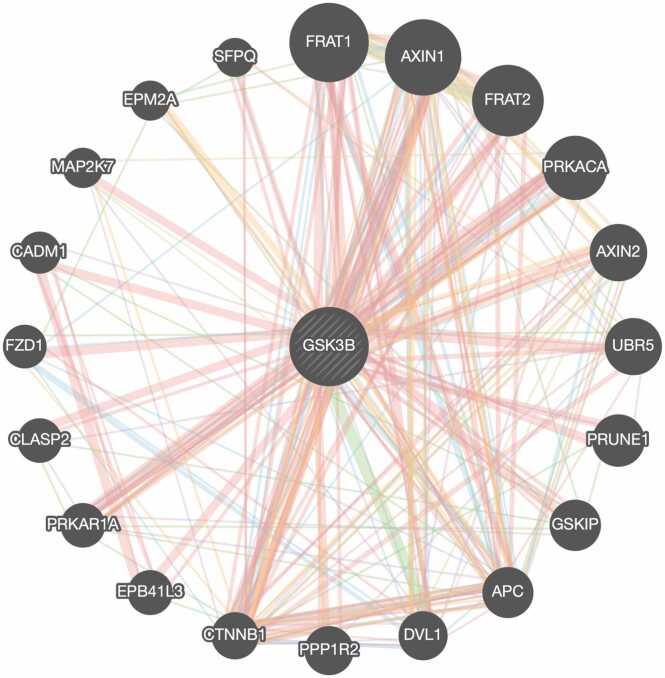
Functional connections between GSK3B and its associated genes. Pink signifies physical interactions, purple suggests co-expression, orange shows predicted interactions, blue implies co-localization, green represents genetic interactions, light blue signifies pathways, and yellow indicates shared protein domains (Created from http://genemania.org/).

The analysis of gene-gene interactions indicated that the GSK3B gene is associated with 20 genes, resulting in a total of 294 connections (Fig. 1). The KEGG Pathway database indicates that the GSK3B gene participates in 48 distinct biological pathways. Six of these pathways pertain to neurodegeneration and neurological processes, with their descriptions provided in Table 1.

**Table 1:** Neurological molecular pathways associated with the GSK3B gene according to KEGG pathway database.

**KEGG Pathway Number**	**Pathway Name**	**Related Neurological Processes**
map04360	Axon guidance	Axon outhgrowth, Axon repuision, Axon attraction
map04722	Neurotrophin signaling pathway	Axonal outhgrow, Axonal guidance, Synapse formation, Axon patterning, Plasticity, Long-term potantiation
map04728	Dopaminergic synapse	Synaptic plasticity
map05010	Alzheimer disease	Axonal transport defects, Mitochondrial dysfunction, Apoptosis, Neurofibrillary tangles
map05020	Prion disease	Axonal transport defects, Neurotransmitter oxidation, Alteration of neuronal function
map05022	Pathways of neurodegeneration - multiple diseases	Ubiquitin-proteasome system distruption, Neuronal disfunction, Axonal transport defects, Neurofibrillary tangles, Mitochondrial dysfunction, ER stress, Alzheimer disease, Huntington disease, Parkinson disease, Amyotrophic lateral sclerosis, Prion disease, Spinocerebellar ataxia

### 3.2 *Analysis of Harmful SNPs*

After doing a search via the NCBI dbSNP database, it was discovered that the GSK3B gene has a total of 80 missense SNPs, 27 of which are considered to be harmful. Among these 27 detrimental SNPs, the SIFT and PolyPhen-2 algorithms both agreed that ten of them were harmful. Table 2 has a comprehensive account of the findings. An investigation into the structure and stability of proteins was carried out using these ten detrimental SNPs, which were identified by MutPred2 scores that were more than 0.7.

**Table 2:** Multiple computational tools have been used to determine in silico functional predictions of missense SNPs in the GSK3B gene. SIFT, PolyPhen scores indicate the predicted effect of amino acid substitutions on protein function, while I-Mutant predicts changes in protein stability. MutPred2 scores suggest the likelihood of structural/functional changes.

				**SIFT Results**		**Polyphen Results**		**I-Mutant results**		
**SNP**	**Aminoacid Change**	**Transcript ID**	**Protein ID**	**Score**	**Prediction**	**Score**	**Prediction**	**Reliability Index**	**Stability Result**	**Mutpred2 Score**
rs140668532	V304F	ENST00000264235	ENSP00000264235	0.027	Deleterious	0.994	Probably Damaging	7	Decrease	0.915
rs200373768	R278G			0.017	Deleterious	0.979	Probably Damaging	8	Decrease	0.910
rs201450363	I182T			0	Deleterious	1000	Probably Damaging	9	Decrease	0.917
rs201871343	L359F			0.036	Deleterious	0.981	Probably Damaging	8	Decrease	0.900
rs374033612	P357A			0.011	Deleterious	1000	Probably Damaging	9	Decrease	0.771
rs140668532	V317F	ENST00000316626	ENSP00000324806	0.033	Deleterious	0.996	Probably Damaging	9	Decrease	0.898
rs200373768	R278G			0.014	Deleterious	0.987	Probably Damaging	8	Decrease	0.896
rs201450363	I182T			0	Deleterious	1000	Probably Damaging	9	Decrease	0.906
rs201871343	L372F			0.036	Deleterious	0.989	Probably Damaging	8	Decrease	0.891
rs374033612	P370A			0.011	Deleterious	1000	Probably Damaging	9	Decrease	0.762

### 3.3 Protein Structure and Stability Analysis

The influence of 10 SNPs, deemed detrimental by both SIFT and PolyPhen-2 software, on protein stabilization was evaluated using I-Mutant 2.0 software. Analysis of the results revealed that 28 of these SNPs reduced protein stability. The comprehensive results are displayed in Table 2.

### 3.4 Protein Function Analysis

Each amino acid possesses distinct characteristics, including charge, size, and hydrophobicity. An analysis of these values indicates that the original wild-type residue and the newly inserted mutant residue often exhibit differences in these characteristics. Table 3 presents models of harmful SNPs in the GSK3B gene.

**Table 3:** Conservation analysis and structural context of harmful SNPs in GSK3B are shown, including residue conservation scores, secondary structure localization, solvent accessibility, and functional domain mapping. These annotations help assess the potential structural and functional impact of mutations [28]. (Created with HOPE software).

### 3.5 Molecular Docking

The impact of potential harmful mutations identified for GSK-3β on the inhibition of this protein was investigated using in silico methods. The interactions of WT and GSK-3β proteins with the VAL304PHE, ARG278GLY, ILE182THR, LEU359PHE, PRO357ALA, VAL317PHE, LEU372PHE, and PRO370ALA mutations with the highly selective inhibitory ligand PF04802367 (PDB ID: 6QH) were examined. The binding affinity of this ligand with wild-type GSK-3β was shown to be −7.2 kcal/mole (Table 4).

**Table 4:** Vina scores and contact residues of PF04802367 with GSK-3β.

**Fig. 2:**
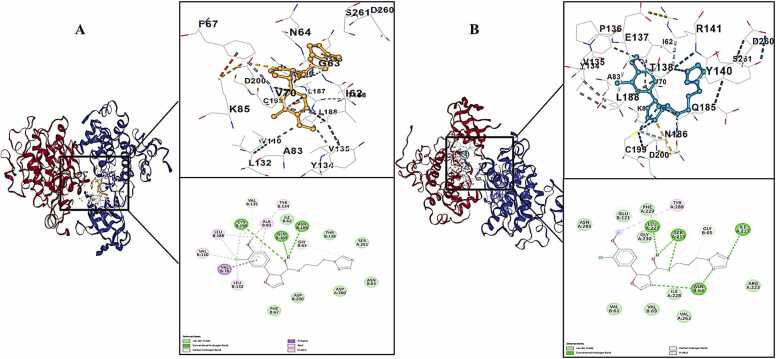
WT (A) and VAL317PHE mutated GSK-3β (B) interactions with PF04802367.

The sequence of modified GSK-3β according to binding energy ranging from −7.1 to −6.4 kcal/mole for the PF04802367 ligand is as follows: R278G= P357A=L372F > L359F=V304F > P370A=I182T > V317F. The current research demonstrates that the maximum binding energy (lowest binding affinity) against the VAL317PHE altered GSK-3β is −6.4 kcal/mole (Fig. 2).

**Fig. 3:**
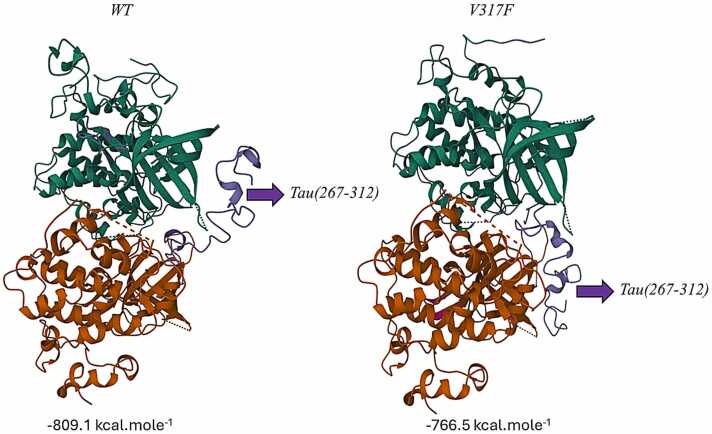
WT and VAL317PHE mutated GSK-3β interactions with Tau(267−312).

Fig. 3 presents the interaction map and binding energies (kcal.mole-1) derived from the protein–protein docking between Tau(267−312) and the VAL317PHE altered GSK-3β. The protein-protein interaction data indicate that the binding affinity of the mutant GSK-3β to Tau(267−312) is inferior to that of the wild type. This outcome is also congruent with the ligand–protein docking findings.

### 3.6 Molecular Dynamics (MD) simulation

MD simulations significantly aid in the analysis and verification of the stability of the anticipated ligand binding mechanism. To assess the mechanical stability of protein-ligand and protein-protein complexes under dynamic conditions, we conducted a 1000 ps molecular dynamics simulation.

**Fig. 4:**
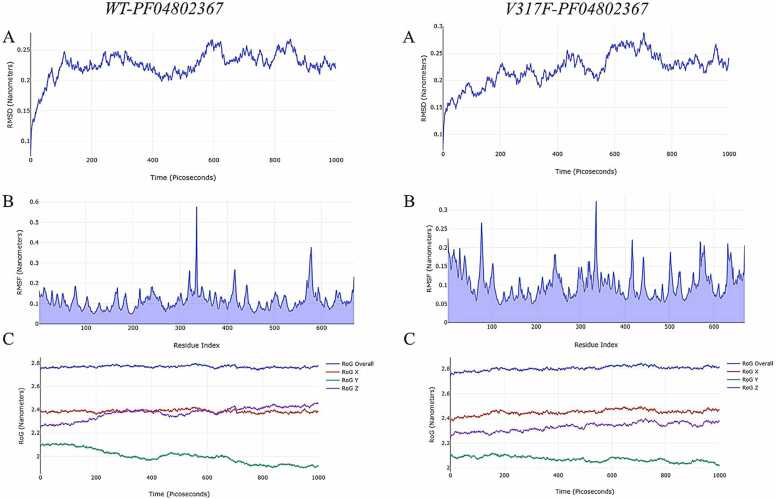
MD simulation results of WT-PF04802367, and VAL317PHE-PF04802367 complexes.

The structural change of a protein from its initial configuration to its final disposition was assessed using the RMSD (Root Mean Square Deviation). Reduced variation correlates with increased stability, while a lower RMSD signifies enhanced stability of the complex [28]. Fig. 4 presents the RMSD results for the WT-PF04802367 and VAL317PHE-PF04802367 complexes. The WT-PF04802367 complex exhibited a consistent range of 0.008 Å to 0.267 Å throughout the 1000 ps molecular dynamics simulation. The VAL317PHE-PF04802367 complex exhibited a consistent range of 0.088 Å to 0.290 Å throughout the MD simulation. The observation that these distances are below 3 Å indicates that the combinations maintained stability during the protein simulation.

Higher RMSF values indicate residues with greater flexibility or motion, while lower values reflect more stable regions [26], [27]. The results demonstrate that the WT-PF04802367 complex shows enhanced stability relative to the VAL317PHE-PF04802367 complex. The scatter plot illustrates the radius of gyration (RoG) as a function of simulation time. A reduced RoG indicates a more compact configuration [26], [27]. There are no notable changes between the WT-PF04802367 and VAL317PHE-PF04802367 complexes.

**Fig. 5:**
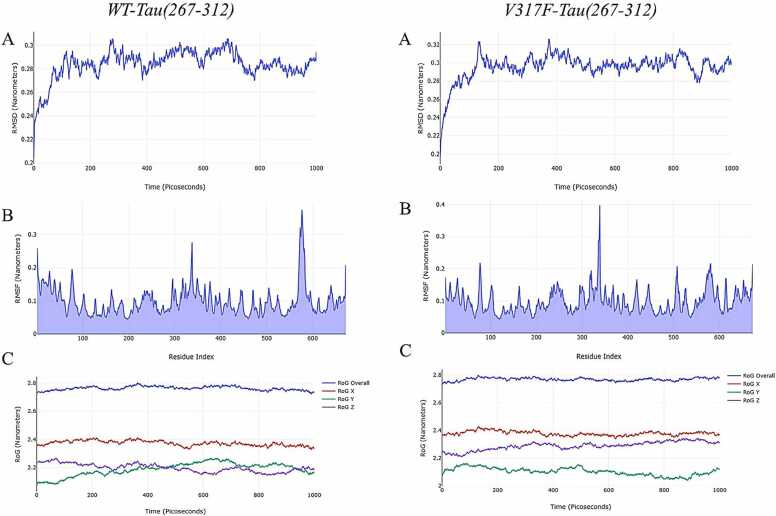
MD simulation results of WT- Tau, and VAL317PHE- Tau complexes.

On the other hand, MD simulation results of the interaction of WT and V317F mutated GSK-3β with Tau are shown in Fig. 5. RMSD results of VAL317PHE mutated GSK-3β-Tau complexes were more than 3 Å in general. When this result is compared with the WT-Tau complex, it is seen that the stability of the GSK-3β-Tau complex is significantly reduced.

## 4 Discussion

The GSK-3β gene plays a key role in glycogen synthesis and is critically involved in several physiological processes, including apoptosis, mitochondrial function, and the regulation of gene transcription. The hyperphosphorylation of Tau protein leads to microtubule destabilization in neuronal cells, thereby impairing cytoskeletal organization and function. GSK-3β is one of the main tau kinases that plays a key role in neurofibrillary tangle formation [29]. Studies have shown that upregulation in the expression of GSK3β in forebrain neurons leads to tau hyperphosphorylation and neuronal death in mice [30], [31]. Several studies have reported associations between polymorphisms in the GSK3B gene region and AD [32], [33].

20 genes directly related to the GSK-3β gene, as identified with GeneMANIA database, were analyzed using the Genecards and NCBI gene databases to determine their roles in specific cellular pathways. Among these, FRAT1, AXIN1, FRAT2, AXIN2, GSKIP, APC and FZD1 genes were found to be involved in the Wnt signaling pathway [34], [35], [36], [37], [38], [39], [40], [41]. The DVL1 gene has a role in neuroblast specification [42]. The EPB41L3 gene has a role in nervous system development and the localization of proteins to the paranodal area of axonal processes [43]. The CLASP2 gene is significant in the development of the neurological system and in autism spectrum disease [44]. The CADM1 gene encodes a synaptic cell adhesion molecule [45]. The high-risk SNPs revealed in our analysis suggest that the disruption of the GSK-3β gene's interaction with its directly associated genes would result in diverse cellular diseases.

As a result of the analysis of the KEGG Pathway database, it was determined that the GSK-3β gene is involved in 6 pathways related to neurodegeneration and neurobiological processes. These pathways directly regulate molecular processes such as axon outgrowth, axon repulsion, axon attraction, synapse formation, axon patterning, plasticity, long-term potentiation, axonal transport defects, mitochondrial dysfunction, apoptosis, neurofibrillary tangles, neurotransmitter oxidation, alteration of neuronal function. As a result of disruptions that may occur in these pathways due to dysfunctions in the GSK-3β gene, neurological diseases such as AD, PD, Huntington disease, ALS, prion disease, spinocerebellar ataxia and schizophrenia may occur.

Among the 10 identified harmful SNPs, the rs140668532 variant is located near a highly conserved site. The variants rs200373768, rs201450363, and rs200373768 are located within an essential protein domain that is critical for its function. The residues are in direct contact with another domain known for its role in binding. The wild-type residue was situated within the protein core in both rs201871343 instances, while the mutant residue is larger and likely incompatible. The novel residue in rs374033612 is situated within the core of a domain, and the mutation may disrupt the distinct conformation of this domain.

The impact of amino acid alterations resulting from the identified SNPs on the GSK-3β protein was examined through molecular docking and molecular dynamics modeling techniques. The V317F mutation associated with rs140668532 diminished the interaction and stability between GSK-3β and PF04802367, as indicated by the results. The interaction between the V317F mutant GSK-3β and Tau led to a reduction in binding affinity and stability for Tau.

GSK-3β enhances the activity of Bax, a pro-apoptotic protein, while inhibiting transcription factors that protect cells from toxic insults [42]. It further enhances the activity of β-secretase (BACE1) and intensifies the toxicity of Aβ aggregates. Inhibiting GSK-3β reduces BACE1-induced cleavage of APP, leading to a reduction in Aβ generation [43]. This kinase is chiefly accountable for Tau phosphorylation. The accumulation of hyperphosphorylated Tau is an additional pathological marker of Alzheimer's disease [44]. Conversely, it phosphorylates PS1, which inhibits APP cleavage and Aβ synthesis while modulating the Aβ 42/40 ratio, hence aggravating Alzheimer's disease [45].

## 5 Conclusion

The V317F mutation's impact on GSK-3β inhibition reduction and the GSK3β-Tau interaction were demonstrated through in silico analysis. The effects of GSK-3β on Aβ formation indicate that the V317F mutation exerts a tau-independent neurodegenerative effect. The experimental investigation of the V317F mutated GSK-3β's effect on neurodegeneration may enhance the understanding of the biomarker potential of rs140668532 in Alzheimer's disease.

## Author statement

Study conception and design: BAA; data collection: IGA, BAA; analysis and interpretation of results: IGA, BAA; draft manuscript preparation: IGA,BAA. All authors reviewed the results and approved the final version of the manuscript.

## Declaration of Competing Interest

The authors declare that they have no known competing financial interests or personal relationships that could have appeared to influence the work reported in this paper.

## Data Availability

Data will be made available on request.
